# Supplementary material for: Effect of Dietary Restriction on Gut Microbiota and Brain–Gut Short Neuropeptide F in Mud Crab, Scylla paramamosain
Source: Animals (Basel). 2024 Aug 20;14(16):2415. doi: 10.3390/ani14162415 (PMC11350653; doi:10.3390/ani14162415)
Supplement: Supplementary file 1 [file animals-14-02415-s001.zip › Supplementary information.pdf]

Supplementary information

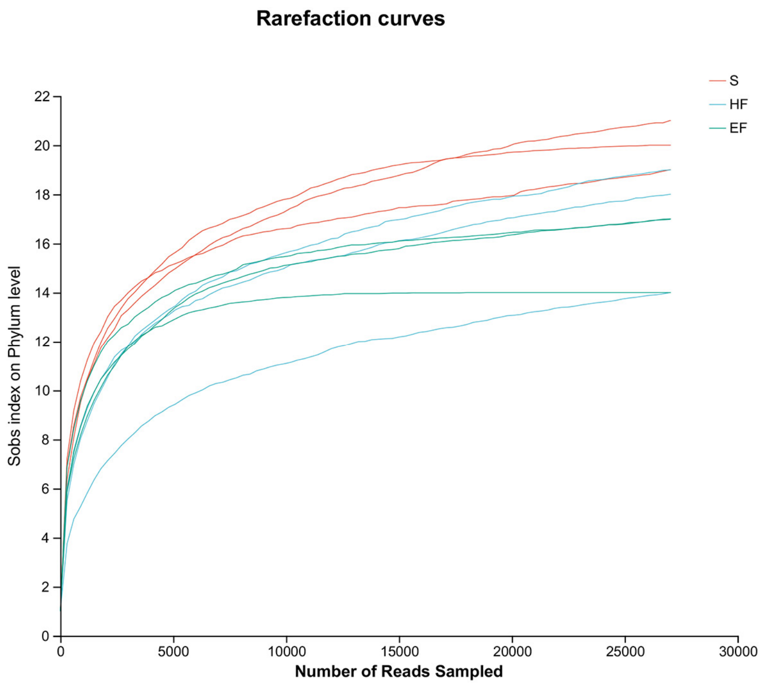

Figure S1. Rarefaction curves for all samples. S, starvation group; HF, 1/2 food group; EF, enough-food group.

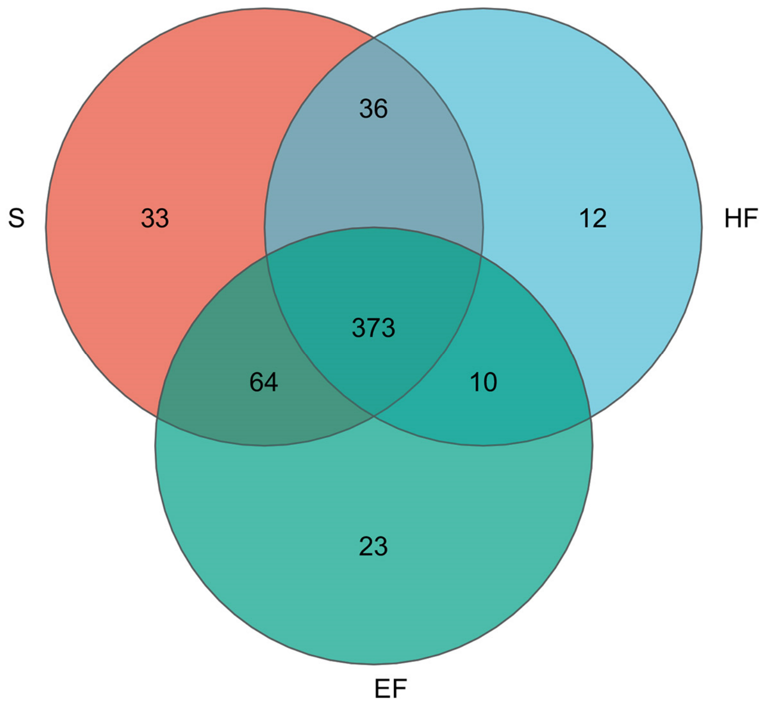

Figure S2. Venn diagram analysis of microbial communities in the gut of mud crabs. See legends of Fig. S1 for the abbreviation.
